# Is paying bribes worthwhile? Corruption and innovation in middle-income countries

**DOI:** 10.1007/s40821-022-00205-4

**Published:** 2022-02-23

**Authors:** Roberto Iorio, Maria Luigia Segnana

**Affiliations:** 1 Department of Political and Social Studies, Via Giovanni Paolo II, 132, 84084 Fisciano, SA Italy; 2 Department of Economics and Management, Via Inama 5, 38100 Trento, Italy

**Keywords:** Corruption, Bribery, Emerging markets, Innovation, Endogenous switching, O12, O31, P27, P51

## Abstract

Corruption research in economics has a long history. Seminal early articles, and older findings contrast with newer developments which have as yet not been measured empirically; in particular the link between corruption and innovating activities suffers from multiple results, on both a national country and company or firm level. This paper examines the corruption-innovation link in transition and emerging countries as the decision to corrupt, and the ability to innovate may not be independent. An endogenous switching regression model is advocated as a suitably methodological way of modeling the joint determination of a firm’s innovation and possible bribes as it implies not only a selection between corrupted and non-corrupted firms, but also heterogeneous effects on innovative activities. The paper shows that, when the selection effect is adequately considered, different firms’ strategies arise. In particular, the treatment effect of corruption on innovation is positive for corrupting firms and negative for non-corrupting firms. Corrupting firms appear rational because paying bribes increases their innovative activities. However, non-corrupting firms also appear rational because in the presence of bribes, their innovating activities would be fewer. Thus, when the selection effect is adequately considered, the effects of so-called “greasing-and-sanding-the-wheels” can co-exist. Finally, the role of competition is also considered. Building on these results, future research can move forward to re-examine economic outcomes such as the productivity or the economic impact of corruption, in the presence (or absence) of selection processes.

## Introduction

The World Economic Forum reports that corruption costs the world economy five per cent of global GDP, approximately $2.6 trillion per year. The World Bank estimates that businesses and individuals pay more than $1 trillion in bribes each year. Corruption is difficult to measure and even though the source of and methodology underpinning these widely–cited statistics are not entirely clear (Wathne & Stephenson, 2021), corruption is often self-sustaining and fosters a corrosive culture of impunity. Recent results do not show significant improvements: the Corruption Perceptions Index from Transparency International reveals that in 2020 the majority of countries experienced little or no improvement in tackling corruption since 2012 and that the COVID-19 crisis was also a corruption crisis. Using a scale of zero (highly corrupt) to 100 (very clean), more than two-thirds of countries score below 50, with an average score of only 43. In some middle-income countries in Asia, the Global Corruption Barometer (GCB) shows that citizens are well aware of the corruption across the region: three-quarters of respondents believe that government corruption is a big problem in their country.

The causes and effects of corruption have been a topic of debate for the last 50 years. It includes various actions associated to large-scale (political) and petty (administrative) corruption, to organized and disorganized corruption. Among these actions, bribery remains the most common in practice, usually in the form of small cash payments to public officials to influence or speed up their actions. In any case, no definition of corruption is completely clear-cut. It can also be interpreted in different ways by varying time and place as well as by discipline.^1^ Among many connotations, corruption is identified as “an illegal or unauthorized transfer of money or an in-kind substitute” (Rose-Ackerman, 1975, p. 187) and “corruption is the abuse of an entrusted power for private gain” (Rose-Ackerman & Palifka, 2016, p. 9) or “corruption is an outcome—a reflection of a country’s legal, economic, cultural and political institutions” (Svensson, 2005, p. 20). However, corruption often occurs where private wealth and public power overlap or in some cases corruption is interchanged with bribery (as in Krammer, 2019). In this paper we mostly confine ourselves to the application of the term corruption, used here as synonymous of bribery^2^.

There is a general consensus on the negative cross-country effects of corruption on economic growth (Campos et al., 2010) but its implications for firms and firms’ strategies are still insufficiently understood (Cuervo-Cazurra, 2016), especially for innovating activities. More precisely, although the connection between innovation and corruption has been ubiquitous, scholars have yet to establish the exact nature of this relationship. Some researchers have found that corruption can boost innovation via removing the rigid obstacles to investment and foster innovation which eventually greases economic growth. Conversely, others have demonstrated that corruption may deter innovation levels and the adverse relationship between corruption and innovation can slow down economic growth.

In transition and emerging markets, corruption remains a ubiquitous feature of doing business. Al Bulushi (2019) shows that corruption may sand the wheels of innovation inputs, yet, it shows no impact on innovation outputs in that region. Firm-level analysis also shows that bribes have national as well as transnational characteristics as frequently reported in the press. Examples are found in multinationals such as Siemens, Samsung, major pharmaceutical companies, Wal-Mart and others, showing often that multinationals adapt to local corruption patterns.^3^ Cross-border corruption can also affect FDI, shift the ownership structure and use local partners strategically (Javorcik & Wei, 2009). At the same time, innovating firms in emerging countries face significant corruption pressure: innovators pay more bribes than non-innovators and are often the target of rent-seeking activity (Ayyagari et al., 2014). So transition and emerging markets appear as appropriate environments for studying firms’ responses to corruption (Cuervo-Cazurra, 2016) also because it affects how firms enter into markets (Dreher & Gassebner, 2013), their management practices (Athanasauli & Goujard, 2015) and the business environment as a whole.

The aim of this paper is to look at the reasons for the coexistence of the grease and sand effect of corruption on innovation in Eastern Europe and the Central Asia region. Reasons are identified in the joint determination of corruptive and innovative activities. To this purpose an endogenous switching regression model is advocated as a suitable way of modeling the joint determination of firm’s innovation and corruption. It allows us to identify the selection effect or the switch from corruption to non-corruption and to show the heterogenous effects (grease or sand) on firms’ innovative activities.

Our initial result indicates that corruption has a positive effect on innovation: we find evidence of the grease effect.

But, when the selection effect is adequately considered, different firms’ strategies arise. In particular, the “treatment effect” of corruption on innovation is positive for corrupting firms and negative for non-corrupting firms. Corrupting firms appear rational because paying bribes increases their innovation activities. Non-corrupting firms also appear rational because in the presence of bribes their innovating activities seem fewer. Corrupting firms pay bribes because they are capable of exploiting the advantages of corruption on innovation. Non-corrupting firms do not pay bribes because there is no effect of corruption on innovation activities. In the first case, corruption greases the wheels of innovation; in the second it sands the wheels of innovation. On a firm-level, this result explains the coexistence of the grease and sand metaphor, as for both corrupting and non-corrupting firms, it can be rational to pay or not to pay bribes.

The endogenous switching technique^4^ identifies the selection effect using micro-survey data to construct counterfactual scenarios of the corruption-innovation relationship for firms of emerging countries.

This study contributes to the literature in the following way. First, it expands the body of work on the causes of corruption and the strategies of how firms adapt to corrupt environments, given that the relationship between corruption and firm-level innovation has only recently received attention. Second, it broadens the results on the effects of corruption, by suggesting that it can affect firms in transition and emerging markets differently, thereby augmenting recent evidence on the strategic use of corruption (Iriyama et al., 2016) for innovation purposes. Third, through counterfactuals this study reveals the coexistence of heterogeneous effects, namely grease and sand, on a micro-level.

The innovation responses of firms are estimated on the basis of a selection between corrupting and non corrupting firms; the heterogeneous results explain the coexistence of grease and sand effects when firm-level data are utilized. In other words, they explain why, in relation to the literature that utilizes aggregate data, grease and sand results coexist.

Finally, this work advances our understanding of better manageable policies (OCSE, 2020) aimed at dismantling systemic corruption. These need to be fine-tuned against hidden governance structures on a country and firm-level, in order to align formal and informal exchanges and regulations.

This paper is structured as follows. Section 2 presents a literature review on the impact of corruption on innovation. Section 3 highlights the main hypotheses. Sections 4 and 5 provide information on data and methodology applied in this research study, and Sects. 5 and 6 give the estimation strategy and the empirical results. Conclusions are presented in the final section.

## On innovation and corruption

Corruption research in economics has a long history, dating back to the rent -seeking literature and seminal early articles including Rose-Ackerman (1975), Shleifer & Vishny (1993) and Mauro (1995). A number of influential and frequently cited surveys on the economic analysis of corruption as well as handbooks have appeared in this field which provide details on the subject. Yet empirical studies on the economic impacts of corruption show very mixed results. Recently, Dimant and Tosato (2018) have compared empirical work on the causes and consequences of corruption, contrasting recent with older findings and surveying newer developments which have not been measured empirically previously. The result of their research is to show that the empirical results of older and more recent studies contradict each other, a contradiction attributed to a number of factors. These include “noisy data” namely new phenomena, different econometric approaches and more extensive data sets. The results suggest that, before being able to settle the debate of conflicting empirical findings and answer the several still-open questions, there is still a long way to go.

In search of the effects of corruption (there is less literature on the effects of growth on corruption) one branch of the literature depicts corruption as “sanding–the-wheels” of growth and development through additional costs and uncertainties as well as inefficient public provisions. In contrast, an opposite view argues for the positive effects (“greasing-the-wheels”) of corruption, especially in weak institutional settings where the costs of preventing corruption can well exceed the expected benefits. Both branches have a long history and a new life. Of course, which of the two views provides a better description of how the world actually works cannot be settled purely on theoretical grounds; it is an empirical matter.

We can now narrow our attention to three aspects of the corruption-innovation link: the role of corruption and innovation for growth and development and the connection between the two.

On the corruption side, there is a vast, empirical literature that explores the links between corruption and economic growth, measured by a range of indicators (GDP, total factor productivity growth, investment rates). Many scholars have tried to look for a relationship between growth in real GDP per-capita and cross-national measures of perceived corruption deriving from surveys of risk analysts, business people, and citizens in different countries. Some of the past research (Mauro, 1995; Mo, 2001) reports empirical evidence that, albeit not particularly robust in that on average, corruption reduces growth. Anokhin and Schulze (2009) also argue that in corrupt environments firms are less likely to benefit from direct foreign investment by companies employing sophisticated technologies. Using data from 64 countries relative to the 1996–2002 period, the authors show there is a positive concave relationship between the control of corruption and the amount of domestic innovation. However, the majority of cross-country literature finds no evidence in favor of the “greasing-the-wheels-hypothesis” as Campos et al. (2010) show using meta-analysis.

All the same endogeneity remains a significant challenge together with conditionality, especially when considering the specific subset of different countries. Conditionality is crucial: indeed, it is repeatedly found that corruption is less detrimental in countries where the institutional framework is weaker and may even be positively associated with efficiency in countries where institutions are extremely ineffective (Méon & Weill, 2010). Again the empirical evidence on corruption, growth and input misallocation dynamics for Central and Eastern European countries shows that the link between changes in corruption and input misallocation is positive and dependent on the geographical, institutional and political context. This is because the smaller the country, the lower the degree of political stability and of civil liberties, and the weaker the quality of its regulations (Gamberoni et al., 2016).

On the innovation side, there is a broad and well-known theoretical and empirical consensus about the major benefits of innovation for economic growth. But, as in the case of the challenges beyond corruption and growth, it is this link between corruption and innovation, albeit under-researched, that has generated the two contrasting results: “sanding-the-wheels” versus “greasing-the-wheels”, with corruption detrimental (beneficial) to innovation, especially in the context of a strong (weak) institutional environment. The evidence available on the effects of corruption on innovation activities has as yet to reach a consensus and its consequences for firms are less understood.

On the one hand, it might be difficult to posit that corruption increases economic growth overall. It follows then that we are in presence of an increasing consensus by the greater part of the literature ultimately supporting the idea that corruption is more likely to impede economic prosperity on a country level, particularly when conditionality is considered. On the other hand, recent scholarly attention has focused on examining the consequences for firm strategies especially on innovative activities, as it is very unlikely that firms are uniformly affected by corruption in different institutional contexts: thus the positive and negative link between innovation and corruption may coexist.

The reactions of firms to corrupt practices differ significantly across countries, depending on the existing institutional environment as well as each firm’s specific characteristics. This can apply to firm’s innovation responses to corruption for which both country context and firm characteristics play a role. For example, corruption has a substantially negative relation with the quantity and quality of innovation thereby reducing innovation output both on average and for the most innovative firms in the US (Ellis et al., 2020). Yet corruption may help innovators in transition or emerging markets with a positive relation possibly modified by the quality and the characteristics of institutions in situ (Krammer, 2019); in developing and emerging economies benefitting both SMEs and large firms in most of their innovative activities (Faraz Riaz & Cantner, 2020); in lower-middle income economies, where corruption reduces the likelihood of a firm adopting innovation (Bukari & Anaman, 2021).

### Corruption and innovation. Heterogeneous effects on a firm-level

Although there is abundant literature on corruption’s effects on firms’ performance and investment decisions, the relationship between corruption and firm-level innovation has only recently received attention with a number of comparative and single-country studies. Scholars seem to agree that corruption affects the degree of innovation employed by firms. One common results is that it is very unlikely that firms are uniformly affected by corruption so that heterogeneous effects are apparent. The reasons for this heterogeneity are found firstly in the different characteristics of innovators and secondly in the different incentives (innovation-discouraging or innovation-enhancing) for politically connected firms.

First, heterogeneous effects can be related to different characteristics of innovators and non-innovators affected differently by public rent-seeking. This result has its theoretical roots in Murphy et al. (1993), who wonder why rent seeking activities are so costly for growth. The characteristics of rent-seeking activities (increasing returns leading to multiple equilibria, bad and good depending upon the relative returns of rent-seeking over productive activities) as well as the characteristics of innovators (innovators are more vulnerable to public corruption than established firms, victims rather than perpetrators) may lead to an uneven balance and a sharp reduction in economic growth.

Secondly, heterogeneous effects are also associated with the impact of political connections that may support or undermine innovative activities in varying contexts as well as creating different reallocation incentives. Since the publication of the seminal paper by Faccio (2006), a literature has been produced on the value of political connections, on asking whether firms that have politicians on the board or as owners perform differently. Nonetheless, establishing the relevant political connections is far from straightforward and measures and methods can vary widely.

A first example based on more than 6000 Chinese firms suggests that politically connected firms tend to have more innovation than non-connected firms, with the significant and positive innovation-enhancing value of political connections, especially for non SOE (State-owned enterprises) and low-tech firms (Su et al., 2019). However the effects of political connections are not necessarily uniform across different types of innovations, as Krammer & Jiménez (2020) show in transition and emerging countries.

A second example are the competition strategies adopted by Italian firms, examined in the presence of political connections. Akcigit et al. (2020) found that firm-level political connections are widespread in Italy, especially among large firms. The results indicate that static advantages of political connections may be associated with dynamic losses and worsening industrial dynamics. This is because resources are reallocated towards connected firms with low incentives to innovate and boost productivity growth. Here, the static benefits of political connection (reduced regulatory or bureaucratic burdens) are evaluated with probable dynamic losses in terms of innovation and growth. Politically connected firms report a higher rate of survival, as well as growth in employment and revenue, but not in innovation and productivity. In this case we have a negative innovation discouraging value associated with political connections.

### Corruption and innovation in transition and emerging countries on a firm-level

Shifting our attention to the specific effect of corruption in transition and emerging markets at the firm level, the starting point, again, is greasing, sanding and their ambiguous effects. Shleifer and Vishny (1993) was one of the first to suggest how costly and warping corruption is in a context of government weakness; nonetheless there are theories supporting the idea that corruption can promote innovation in certain cases (Acemoglu & Verdier, 2000) or that it can be harmful to innovation and economic growth (Rose-Ackerman, 1975). The evidence currently available tends to suggest that the connection between innovation and corruption is complex with possible two-way causality; the impact of corruption on innovation may depend on varied types of innovation and corruption and on local institutions.

Examples of a positive link between corruption and innovation are numerous, when considering dissimilar kinds of corruption (petty and grand), varying types of innovation, different channels and directly observable variables in smaller units of analysis. The East Asian paradox, is a greasing puzzle with high levels of corruption but very fast economic growth; it is a puzzle treated at the micro-level for Chinese firms (Wang & You, 2012), with empirical evidence that corruption enhances the growth of revenue as well as innovating activities by Chinese firms. The same results hold for the Turkish case, especially when local dimensions of corruption are considered.^5^ Nguyen et al. (2016) also find empirical support for the grease effect of petty corruption in Vietnam. Krammer (2019) explores the micro evidence in emerging countries, providing evidence of positive effects of petty corruption on developing new products in transition economies, a link weakened by the quality of the formal and informal institutions in place. Benefits are also identified by Faraz Riaz and Cantner (2020) for most of the innovative activities in lower-middle income economies, where corruption reduces the likelihood of a firm’s adoption of innovation (Bukari & Anaman, 2021). Using data from Egypt and Tunisia to represent the MENA region, where corruption is perceived to be persistently high, Goedhuys-Degelin et al. (2016) test the hypothesis that the link between corruption and innovation depends on the severity of bureaucratic and institutional obstacles. Results show that corruption has a directly negative effect on a firm becoming innovative but a positive effect when it interacts with institutional obstacles. Corruption in this case is simply a mechanism to bypass bureaucratic hurdles, such as business permits and licenses thus lessening weakening the negative impact of obstacles on innovation.

Support for the simultaneous occurrence of the “sanding/greasing the wheels” hypotheses are found in Mahagaonkar (2008) who researched varying kinds of innovation of African firms and is also explained by the divergent effects of the average amount of corruption (Hanousek & Kochanova, 2015).

Though examples of the negative link abound, in many cases the coexistence of the two effects is the most common outcome. Habiyaremye and Raymond (2013) report that foreign firms’ corruption practices in transition economies are detrimental to R&D efforts in the host country. For Ugandan firms too, the result is the detrimental impact of corruption on the growth of firms (Fisman & Svensson, 2007). With different lenses, Ayyagari et al. (2014) conclude that corruption acts as a tax on innovation in developing countries, i.e., innovator firms pay more in bribes as they are more likely to become victims of corruption than perpetrators.

If we match country-level and firm-level results, we can reach a conclusion by observing persistently contrasting findings. In other words, we primarily find on a country level that the institutional environment matters. Any kind of aggregate result must carefully account for country characteristics and conditionality; secondly, the persistence of the mixed sand and grease results on a firm level go hand by hand with the heterogeneous impact of corruption on innovation.

There are many reasons for these micro mixed results: undeniably micro firms’ characteristics play a role together with the macro institutional environment as well as the differing methods applied to survey and longitudinal data. The struggle to understand the multifaceted nature of corruption and its interactions on the micro, meso, and macrolevel, first requires us to focus on microdata. We need to do this to overcome the shortcomings of cross-country macro-data and also to define the correct way of measuring corruption in observational as well as in experimental data.

## Bringing the main hypotheses to data

From our comparisons, we have developed three main hypotheses.

First, the institutional environment matters. In their efforts to control corruption, countries with weak institutions enhance the ability of bribes to function as efficient grease. In contrast, a strong regulatory control of corruption significantly reduces the ability of bribes to facilitate the introduction of innovation, thus reducing kick-backs for colluding bureaucrats and reducing bribery’s power to achieve the desired outcome.

Second, there is a heterogeneous effect of corruption on different firms, e.g. on innovators versus non-innovators. This differentiation mirrors a selection effect across countries and between firms that adapt to corruption and firms that do not. This effect might, on one hand, be explained by the country effect and on the other by the specific characteristics of firms.

Third, the selection effect acts differently on the inclination to innovate. Corrupting firms pay bribes because they can exploit the advantages of corruption on innovation. Non-corrupting firms do not pay bribes because there is no effect of corruption on new, innovative activities.

Bringing these hypotheses to data implies that we can proceed with the following empirical tests:

- H1. On average, corruption has a positive effect (greasing-the-wheels) on the innovative activities of firms in emerging and transition markets.
- H2. There is a selection effect that differs between corrupting and non-corrupting firms. This selection effect is due mainly to the observable characteristics of a country and its firms’ characteristics as well as non-observable characteristics. They both motivate the decision to pay a bribe and the firm’s ability to innovate.
- H3. Taking selection and a firm’s heterogeneity into account, we can discern a two-fold impact of corruption on innovation namely the coexistence of both greasing and sanding impacts, affecting different clusters of firms.

These hypotheses have been using data from more than 17,000 firms in 36 emerging markets in Central and Eastern European, Central Asia and some North African countries. They reveal substantial heterogeneity in terms of corruption practices, institutional quality and innovative potentials.

## Data sources and descriptive statistics

We have employed firm-level data on innovation and corruption from the BEEPS V-MENA ES (in the following: BEEPS),^6^ firm-level survey promoted by the European Bank for Reconstruction and Development, the World Bank group and the European Investment Bank. This dataset covers 36 emerging markets, including the more advanced Central and Eastern European countries, transition economies from the Balkans and Central Asia as well as Turkey and the nations of the South and East Mediterranean. Interviews were conducted between 2012 and 2014. The final sample consisted of 17,133 firms.

The BEEPS survey is conceptually rigorous and avoids measurement biases: it employs standardized survey instruments and stratified sampling techniques^7^ (on the level of the two-digit International Standard Industrial Classification code ISIC Revision 3.1, firm size, and geographical location). Descriptive statistics and an overview of the variables are reported in Table 1 and in the Appendix.

**Table 1:** Description and statistics of the variables

Variable	Description	N	Mean	SD
innoprod	Product innovation in last three years (0/1)	17,072	0.25	0.43
innosales	Innovative sales as a percentage of annual sales	16,280	6.74	18.37
innosales_onlypositive	Innovative sales as a percentage of annual sales (only firms with positive *innosales*)*	3481	31.51	28.24
innosales_mean	Mean of *innosales* by country	17,133	6.75	2.67
bribe	Informal payment “to get things done” as a percentage of annual sales	13,678	0.66	3.61
bribe_yes	Informal payment “to get things done” (0/1)**	13,678	0.10	0.30
bribe_onlycorrupt	Informal payment as a percentage of annual sales (only firms paying bribes)***	1400	6.45	9.48
bribe_mean	Mean of *bribe* by country	17,135	0.82	0.99
R&D	R&D expenditure in the last three years (0/1)	17,031	0.11	0.32
fortech	Licensed foreign technology (0/1)	16,992	0.12	0.33
train	Formal training for permanent emplyees (0/1)	16,993	0.32	0.47
ncomp	Number of firm’s competitors, more (less/equal) than five (0/1)	17,133	0.74	0.44
dmon	Competitors, more(less) than 1 (0/1)	14,436	0.04	0.19
size	Natural logarithm of full-time employees	17,069	3.14	1.32
employees	Number of full-time employees	17,069	80.66	330.81

*For firms declaring a positive percentage of innovative sales on annual sales (firms having *innosales* >0) the value of this variable is equal to *innosales*; all the other firms have a missing value for this variable

**This variable assumes value 1 for firms declaring a positive percentage of informal payment “to get things done” on annual sales (firms having *bribe* > 0); it assumes value 0 for firms declaring a percentage of informal payment “to get things done” on annual sales equal to 0 (firms having *bribe* = 0); all the other firms have a missing value for this variable

***For firms declaring to have paid an informal payment “to get things done (firms having *bribe_yes* = 1) the value of this variable is equal to *bribe;* all the other firms have a missing value for this variable

### Variables, bivariate and multivariate statistics

Corruption is the determinant and innovation is the dependent variable. The variables concerning corruption are drawn from the answers to J7 question of the BEEPS questionnaire: “*It is said that establishments are sometimes required to make gifts or informal payments to public officials to “get things done” with regard to customs, taxes, licenses, regulations, services *etc*. On average, what percentage of total annual sales, or estimated total annual value, do establishments like this one pay in informal payments or gifts to public officials for this purpose*?”. The binary variable *bribe_yes* indicates if a firm met or did not meet such an informal payment; the variable *bribe* indicates the payment amount as a percentage of total sales. To focus attention on the corrupting firms only, the variable *bribe_only corrupt* is created, which is equal to *bribe* but has values only for those firms that did actually pay a bribe and it has missing values in the other cases.

In order to account for innovative activities, we use different empirical specifications: the binary variable *innoprod* indicates whether or not the firm had introduced a product innovation in the previous three years; *innosales* is the percentage of annual sales accounted for by new or significantly improved products; *innosales_onlypositive* is equal to *innosales* with values only for firms that declared a positive value for the percentage of innovative sales and missing values in the other cases. The definitions of all the variables used in the empirical analysis, the number of observations, their average and standard deviations are all reported in Table 1.

The description of the sample is completed by Tables A1 and A2 in the Appendix. These report respectively the number of firms by country and by productive sector.

Some observations can be summarized on the basis of Table 1 and Table A2 in the Appendix.

- A quarter of the firms introduced at least one product innovation.
- The percentage of annual sales accounted for by innovating activity is on average 6.7%, a percentage that increases to 31.5% when innovative firms alone are considered.
- Overall, informal payments reach 10% of the firms. On average corrupt firms pay 6.6% of their annual sales on informal payments to “get the things done”. If we take all firms into consideration, the incidence of such payments is a mere 0.66% of total sales.
- Correlations between innovation and corruption is positive and significant for most variables.

In the bivariate Tables A3 and A4 in the Appendix, we first examine whether innovating firms pay more bribes than non innovators. In particular, 15% of innovators pay bribes versus 8.7% of non innovators. We then look at subsamples of firms that pay bribes. Here innovators pay more. We report finally that overall innovators pay a higher percentage of their sales as bribes.

The results show that innovating firms are disproportionately affected by corruption, thereby confirming that bribe payments are a tax on innovation (Ayyagari et al., 2014) and innovators are more vulnerable to corruption (Murphy et al., 1993). However, we know little from existing literature about whether innovators pay more bribes, because this facilitates the introduction of significant improvements (innovating activities) by bypassing or overcoming bureaucratic obstacles.

The link between corruption and innovation needs to be better investigated through a multivariate analysis. As dependent variables we have considered both *innoprod* and *innosales.* The first one is a binary variable, therefore a probit is utilized. The second one is a percentage, with a conspicuous amount of zero values, therefore it may be considered a bilaterally truncated variable and a Tobit is used. We also report the results of an OLS estimation.

As “corruption” variables we have made use of *bribe_yes, bribe* and *bribe_onlycorrupt*. The latter is equal to *bribe* but has values only for firms that did actually pay a bribe. The analysis is limited to these firms.

The link between innovation and corruption identified by the basic statistics includes both country and firm characteristics and includes a “between” country and a “within” country effect. In order to isolate the importance of the country effect in our comparative perspective, we estimate the link by introducing country dummies in the regression analysis. Such variables explain a high percentage of the variability of the dependent variable, confirming the presence of a strong “between country” effect.

The results, reported in Table 2, show that the coefficients of *bribe_yes* and *bribe* are positive and significant in all cases, pointing to an interesting “in-country” connection with innovation. Only the variable *bribe_onlycorrupt* is insignificant and this indicates that what is important for innovation is whether or not bribing activities have entered into play rather than the amount of the bribe itself.

**Table 2:** Corruption and innovation

Covariates	Dependent variable (model)								
	Innoprod (probit)(1)			Innosales (Tobit)(2)			Innosales (OLS)(3)		
bribe_yes	0.27**			13.11**			2.27°		
Bribe		0.01**			0.63**			0.13	
bribe_onlycorrupt			0.01			0.20			0.02
R&D	0.99**	0.90**	0.69**	43.74**	44.09**	25.52**	12.09**	12.15**	10.49**
Fortech	0.35**	0.35**	0.31**	17.51**	17.65**	10.51*	4.16**	4.19**	2.72
Train	0.32**	0.32	0.41**	15.55**	15.67**	13.08**	2.68**	2.69**	1.68
Ncomp	− 0.012**	− 0.12**	− 0.08	− 8.38**	− 8.13**	− 4.98	− 1.59**	− 1.54**	− 0.29
Size	0.04**	0.04**	0.11**	0.46	0.44	2.97*	− 0.28	− 0.281	0.618
Country dummy	Yes	Yes	Yes	Yes	Yes	Yes	Yes	Yes	Yes
Industry dummy	Yes	Yes	Yes	Yes	Yes	Yes	Yes	Yes	Yes
N	13,346	13,346	1360	12,756	12,756	1306	12,756	12,756	1306
Pseudo R^2^	0.1678	0.1660	0.1632	0.0531	0.0524	0.0547	0.1060	0.1053	0.1207

**p < 0.01 ; *p < 0.05; °p < 0.10

Significant and positive variables are R&D, foreign technology licenses (*fortech*) and training programs (*train*). Significant and negative is the degree of competition (*ncomp*). The size of the firm does not show up in results, probably reflecting an ambiguity: on the one hand we are in the presence of a positive connection between size and innovating activities; on the other and considering innovative firms alone, *size* and *innosales* are negatively linked. This ambiguity, together with the correlation with other covariates, renders variable insignificant (Tobit) or, when significant, gives it a different impact (probit and OLS). We also introduced sectoral dummies, that contribute to explain the variability of the dependent variables.

Results indicate that corruption impacts positively on innovative activities within countries, together with the traditional indicators affecting the propension to innovate (i.e. R&D, training and foreign licenses). On the contrary, competition impacts negatively on innovation as more competition reduces innovative sales in all cases.

## Estimation strategy

The simplest approach to examining the impact of corruption on innovation is estimating a single innovation equation including corruption as a covariate; indeed this was the first step in our analysis. This is a preliminary approach, because it may yield biased estimates as it assumes that adaptation to corruption is exogenously determined, while it is potentially endogenous. The decision to adapt or not to corruption is voluntary and may be based on individual self-selection. Firms that corrupt may have systematically different characteristics from the firms that did not adapt because different expected benefits play a role. The unobservable characteristics of firms may affect both the decision to corrupt and innovation. If this happens, self-selection or endogeneity problem arises, as the predictors of outcomes are associated with the error term in outcome equations, thus generating inconsistent estimates in the effects of corruption on innovation. This is a very common problem in cross-sectional data.

The IV (instrumental variable) method is a widely-used approach for addressing endogeneity and it has been used by many authors that have studied the corruption-innovation link. Matching techniques have also been used for detecting substantial bias in self-selection by examining proper treatment effects. In both cases (matching and IV) a drawback has been identified in the presence of heterogeneity (Blundell & Dias, 2009). For instance, in “heterogeneous” treatment effect models, in which the impact parameter can differ in unobservable ways across individuals, the IV estimator will not generally highlight the average treatment effect unless in the context of strong assumption, ones unlikely to hold up in practice.^8^

In our case, the key concern is to look at the endogeneity between corruption and innovation as well as heterogeneous behavior corrupting and not corrupting firms. The possible occurrence of heterogeneity has also become a major topic in evaluation research in recent years and in our case it would appear as crucial.

The Endogenous Switching Model (ESM from now on) is an application of the control function method (Murtazashvili & Wooldridge, 2016) that directly analyzes the choice problems facing firms deciding to adapt to corruption participation.

The control function approach specifies the joint distribution of the assignment rule and treatment. It uses the specification of the assignment rule together with an excluded “instrument” from which to derive a control function that, when included in the outcome equation, fully checks for endogenous selection. This approach relates directly to the selectivity estimator à la Heckman. In contrast to the Heckman model, in the switching regression approach firms are partitioned according to their (un)corruptive behavior, in order to capture the differential responses of the two groups. Therefore, ESM is an estimation choice which not only accounts for endogeneity, but also checks for possible heterogeneity effects.

An endogenous switching regression model is advocated as a suitable way of modeling the joint determination of a firm’s innovation and bribes. The endogeneity of switching from corruption to non-corruption comes from the fact that the decision to corrupt and the ability to innovate may not be independent. The heterogeneity comes from the fact that a decision to corrupting or not corrupt may imply different effects in the firm’s characteristics and on its innovative activities.

More specifically, in the first stage, a self-selection equation is estimated and, in the second stage, two outcome equations conditional on the treatment (i.e. corruption decision) are modeled.

Hence, this two-stage switching regression model has the advantage of estimating separate regression equations for corrupted and non-corrupted as well as determining the counterfactuals.

The ESM takes the following form:

1 $$\begin{aligned}& bribe\_yes*i = {\varvec{Z}}{\varvec{i}}\boldsymbol{\alpha }+ \eta_\text{i}, \text{ with}\\ & bribe\_{yes_i}=\left\{\begin{array}{l} 1, if { brib{e\_}{yes}*}_{i}>0\\ 0, otherwise\end{array}\right.\end{aligned}$$

where (1) represents the selection equation, ***Z***_**i**_ the determinants of the latent variable bribe_yes*_i_ at the firm-level for the decision to corrupt or not (to pay or not a bribe “to get things done”). This allows us to check and classify selection into alternative regimes (1 for corruption, 2 for non corruption), characterized by different relationships between the independent variables and the dependent one (innovation). The second stage of the model is therefore represented by two outcome equations, having the innovation outcome as dependent variable and different coefficients for the same independent variables.

2a $$\text{Regime 1}: \text{Y}_{\text{1i}}= {\varvec{X}}_{\varvec{1i}} {\varvec{\beta}}_{\varvec{1}} + \varepsilon_{1i}\text{ if }bribe\_{yes_i} = 1$$

2b $$\text{Regime 2}: \text{Y}_{\text{2i}}= {\varvec{X}}_{\varvec{2i}} {\varvec{\beta}}_{\varvec{2}} + \varepsilon_{2i}\text{ if }bribe\_{yes_i} = 0$$

where *Y*_i1_ and *Y*_i2_ is the innovation variable (percentage of sales by innovative products) respectively in regimes 1 and 2, and ***X***_**i**_ represents a vector of inputs affecting innovation performance.^9^

An important implication of the error structure is that, because the error term of the selection Eq. (1) is assumed to be correlated with the error terms of the outcome functions (2a) and (2b), the expected values of the error terms in (2a) and (2b), conditional on the sample selection, are nonzero. If the estimated covariances are significantly different from zero, then the decision to corrupt and the decision to innovate are correlated. This indicates evidence of endogenous switching and the null hypothesis of the absence of sample selectivity bias is not accepted.

The model is estimated by full information maximum likelihood (FIML). An alternative estimation method is the two-step procedure. However, this method is recognized as less efficient than FIML, as it requires some adjustments to achieve consistent and standard errors.

This approach to survey data can be used for a very interesting purpose: comparing actual results with counterfactual ones. In fact, the model hypothesizes that firms behave differently (coefficients are different) in two different regimes. Therefore, once the parameters have been estimated, for the firms that actually corrupted (paid a bribe “to get things done”) it is possible to calculate the expected outcome in both the “corruption” regime and in the “no corruption” regime: the first outcome is the expected outcome in the actually chosen regime, the second one is the counterfactual, or the outcome the corrupting firms would have obtained if they had not corrupted. The difference between the two outcomes is the expected effect of the corruption. The same holds for the firms that did not corrupt: the expected value calculated in the “no corruption” regime is the expected outcome in the actually chosen regime, while the expected value calculated in the “corruption” regime is the counterfactual, namely the outcome they would have obtained if they had corrupted. The difference between the second one and the first is the expected (potential) effect of the corruption.^10^

This approach allows us to go beyond the calculation of an “average” effect of corruption, as the effect may be different in the two groups; among the corrupting firm it has a real effect, among the non-corrupting it has a potential effect. Therefore, these different effects may enlighten the rationality (or otherwise) of the firms. Firms are “rational” if they choose the regime (corruption or no corruption) that, given their characteristics, increase their innovative outcomes.

To sum up, this model has two advantages: firstly with the hypothesis of correlation between the errors in the selection equation and the errors in the outcome equations,^11^ it deals with the endogeneity of the corruption choice; secondly, with the hypothesis of the existence of two different regimes,^12^ it permits us to calculate the effect of the decision variable—corruption—through counterfactuals.

Table 3 summarizes the conditional expectation outcomes of the innovator’s performance. The two subsamples, identified by the actual choice of the firm to corrupt or not to corrupt, (i.e. paying or not paying a bribe to “get things done”) are in rows, while the two different regimes (corruption and no corruption) are in columns.

**Table 3:** Actual and Counterfactual cases

Subsamples	Regimes		Treatment effects
	Corruption	No corruption	
Firms that corrupt	(a) E (Y_1i_ | CORR_i_ = 1)	(c) E (Y_2i_ | CORR_i_ = 1)	TT = (a) – (c)
Firms that do not corrupt	(d) E (Y_1i_ | CORR_i_ = 0)	(b) E (Y_2i_ | CORR_i_ = 0)	TU = (d) – (b)
Heterogeneity effect	BH1 = (a) – (d)	BH2 = (c) – (b)	TH = TT-TU

Cell content can be specified as follow:

- (a) E(Y_1i_|CORR_i_ = 1) is the expected value of *innosales* calculated in the “corruption regime” among the firms that actually corrupted.
- (b) E(Y_2i_|CORR_i_ = 0) is the expected value of *innosales* calculated in the “no corruption regime” among the firms that actually did not corrupt.
- (c) E(Y_2i_|CORR_i_ = 1) is the expected value of *innosales* calculated in the “no corruption regime” among the firms that actually corrupted
- (d) E(Y_1i_|CORR_i_ = 0) is the expected value of i*nnosales* calculated in the “corruption regime” among the firms that actually did not corrupt.
- TT is the effect of the Treatment on the Treated: the difference between (a) and (c), that is the advantage given by the corruption to the firms that actually corrupted respect to the hypothetical situation (the counterfactual) they had not corrupted.
- TU it is the effect of the Treatment on the Untreated, the difference between (d) and (b), that is the potential advantage given by the corruption to the firms that did not actually corrupt (the counterfactual) respect to the actual choice not to corrupt.

Where:

- E (Y_1i_ | CORR_i_ = 1) = β_1_X_1i_ + σ_1η_λ_1i_
- E (Y_2i_ | CORR_i_ = 0) = β_2_X_2i_ + σ_2η_λ_2i_
- E (Y_2i_ | CORR_i_ = 1) = β_2_X_1i_ + σ_2η_λ_1i_
- E (Y_1i_ | CORR_i_ = 0) = β_1_X_2i_ + σ_1η_λ_2i_

being: σ_1η_ the covariance of η_i_ and ε_1i_; σ_2η_ the covariance of η_i_ and ε_2i_; λ_1i_ = φ(**Z**_**i**_**,α**)/Φ(**Z**_**i**_**,α**); λ_2i_ = φ(**Z**_**i**_**,α**)/(1-Φ(**Z**_**i**_**,α**)), where φ (.) and Φ (.) are the standard normal probability density function and normal cumulative density function respectively.

It follows that:

$${\text{TT }} = \, \left( {\text{a}} \right) - \left( {\text{c}} \right) \, = \, (\beta_{{1}} {-}\beta_{{2}} ){\text{X}}_{{{\text{1i}}}} + \, (\sigma_{{{1}\eta {-}}} \sigma_{{{2}\eta }} )\lambda_{{{\text{1i}}}}$$

$${\text{TU }} = \, \left( {\text{d}} \right){-}\left( {\text{b}} \right) \, = \, (\beta_{{1}} {-}\beta_{{2}} ){\text{X}}_{{{\text{2i}}}} + \, (\sigma_{{{1}\eta {-}}} \sigma_{{{2}\eta }} )\lambda_{{{\text{2i}}}}$$

Following Di Falco et al. (2011) we can also use the previous results for calculations of the heterogeneity effect BH1 and BH2 in the table. BH1 is the difference between (a) and (d). BH2 is the difference between (c) and (b). They show the effect of the base heterogeneity for firms that decide to corrupt (BH1) or not to corrupt (BH2). Furthermore, the transitional heterogeneity (TH is whether the effect of corrupting is greater or smaller for firms that actually corrupted or for firm that actually did not corrupt in the counterfactual case that they did corrupt, that is the difference between (TT) and (TU).^13^

Summing up, the empirical strategy suggests a way to use survey data on the corruption-innovation link by identifying the driving forces behind a firm’s decisions to adapt or not to adapt to corruption, and to investigate whether or not these choices impact differently on innovation.

## Results

We posit an Endogenous Switching Model, where the outcome variable is innovation (*innosales*) and the selection variable is corruption (*bribe_yes*)*.* The covariates are the same used in the previous models, except for country and sector dummies, which are excluded by the model due to computational difficulties.^14^ The country effect is registered by *innosales_mean,* and the selection equation also includes *bribe_mean*, the country mean for *bribe*.

Table 4 shows the ESM results: in the first column the results of the selection equation (determinants of *bribe_yes*) are reported; the second and third columns highlight the results of the outcome regressions (determinants of *innosales*) respectively for corrupting and not corrupting firms. In the fourth column we report the results of a linear regression (OLS), with *innosales* as dependent variable and, as covariates, the same included in the two outcome regressions of the ESM, plus the selection variable *bribe_yes*.

**Table 4:** Results of the endogenous switching model

Covariates	Selection equation Dependent Var.: *bribe_yes*	Outcome equations Dependent Var.: *innosales*		“Control” OLS regression Dependent Var.: *innosales*
		Firms with *bribe_yes* = 1	Firms with *bribe_yes* = 0	
*bribe_yes*				2.129°
*R&D*	0.260**	11.502**	12.609**	12.445**
*Fortech*	0.119**	4.330	4.238**	4.215**
*Train*	0.000	1.119	1.842**	1.815**
*Size*	-0.013	0.724	-0.057	0.021
*innosales_mean*	0.032	1.316**	0.880**	0.923**
*Ncomp*	0.145**	0.544	-1.531**	-1.348**
*bribe_mean*	0.495**			
N: 12,769				R^2^ = 0.0861
Wald chi2 (6) = 209.69 (Prob > chi2 = 0.000)				
rho1: 0.067 (p-value: 0.085)				
rho2: 0.011 (p-value: 0.518)				
Wald test of independent equations: chi2(1) = 3.11 Prob > chi2 = 0.0780				

**p < 0.01 ; *p < 0.05; °p < 0.10

The last rows of the column highlight some statistics: the number of observations, the Wald χ^2^; the value of ρ1 and ρ2 (rho1 and rho2): these are the covariance between the error terms of the selection equation with respectively the outcome equation of the corrupting firms and the outcome equation of the non-corrupting firms; the Wald test of independence between the two equations.

Table 5 shows the expected values and the counterfactual results, already identified in Table 3.

**Table 5:** Counterfactual results

(a)E(Y_1i_|CORR_i_ = 1) = 10.410
(b)E(Y_2i_|CORR_i_ = 0) = 6.691
(c)E(Y_2i_|CORR_i_ = 1) = 8.615
(d)E(Y_1i_|CORR_i_ = 0) = 6.192
TT = (a) – (c) = 1.795 (95% confid.interval: lower bound:1.698; upper bound: 1.892)
TU = (d) – (b) = -0.500 (95% confid.interval: lower bound -0.530 upper bound:-0.469)
Heterogeneity effects: BH1 = 4.218BH2 = 1.924TH = TT-TU = 2.295

The main results may now be summarized as follows.

1. Two different “regimes” are identified, determined by the choice to pay or not to pay any informal payments. In the two regimes firms behave differently: the determinants of innovative sales have different effects. Among firms that pay informal payments, besides the country mean for innovative sales, only R&D has a significant link to innovative activity, while among the firms that do not make any informal payments there are several variables, beyond the country effect and R&D, significantly linked with innovation, namely internal training, foreign technology and the number of competitors.
2. The statistical tests (particularly rho1 and the Wald test of independency of the equations) suggest the possibility of an endogenous selection process, which cannot be rejected at a 10% per cent level of significance. This result contrasts with Krammer (2019) where data from the 5th BEEPS survey are analyzed and the relationship between corruption and innovation is identified through the interaction effects with the quality of institutions. In this case, using a maximum likelihood Heckman probit, the selection bias does not appear significant.
3. The effect of the “treatment corruption” on firms that decide to make informal payments, is significantly positive (TT > 0): the firm that pays a bribe performs worse without corruption. The effect of the “treatment corruption” on the firms that decide not to make informal payment is negative (TU < 0) and significant. In this case, firms not paying a bribe would perform worse if they decided to make an informal payment. The behavior of firms therefore appears rational and the corruption is seen as an opportunity (or a need) only for a select group of them.
4. There is a strong country effect, both on a firm’s corruption and on the degree of innovation.
5. The competitive scenario matters. A firm’s inclination to make informal payments increases with the number of competitors. This shows that paying a bribe reduces the negative impact of competition on innovation (among firms not paying a bribe the percentage of innovative sales decreases with the number of competitors, while this relationship is not significant among firms paying a bribe). This result is in line with what Kabadurmus and Sylwester (2020) find using the same data and utilizing a conditionally mixed process model to address endogeneity concerns. In that case, the relationship between corruption and innovation is stronger among firms with many competitors.

In terms of the hypotheses formulated at the end of Sect. 2, we make three final observations.

Firstly, corruption (paying informal payment “to get things done”) does have on average a positive relationship with the probability of introducing product innovation and with the percentage of sales deriving from said innovation. This is consistent with H1: the greasing average effect of corruption on innovation in the countries under inspection.

Secondly, there are signals of a selection effect involving innovation and corruption. Not only do the observable determinants of innovation play a role as determinants of corruption (particularly, the country effect, and the degree of competition), but also have an effect on the connection among the unobservable determinants of both. This is in line with H2 which suggests interactions in common drivers of corruption and innovation.

Thirdly, the selection effect shows that corruption has different effects on two different groups of firms with a positive (negative) effect in terms of innovative activity on those firms that rely (or do not rely) on informal payments. This result is in line with H3, showing that, when both the selection and firms’ heterogeneity are considered, the impact of corruption on innovation can generate both greasing and sanding results.

## Conclusions

Prior research shows that innovators are disproportionately affected by bribing practices as opposed to non-innovators (Ayyagari et al., 2014). However, the heterogeneous effects of corruption on innovation are often identified in the empirical literature. The connection between corruption and innovation is complex with heterogeneous impacts due to two-way causality, differing types of innovation, corruption and local formal and informal institutions in which both, firms and national characteristics, play a role. This is particularly important for the regions under inspection, namely transition and emerging countries.

From a methodological point of view, our results show that the effectiveness of bribery in greasing the wheels of innovative activities could be contingent upon the selection processes between corrupting and non-corrupting firms (the two regimes) across countries. Thus, the most interesting finding of this study is that greasing and sanding may actually coexist in the region examined, depending on the level of analysis, and on whether or not the selection processes play a role.

Selection processes point to a rationality of the corruption-innovation link. In this case, calculating an “average” effect of the corruption does not make much sense: paying or not paying a bribe is not an accident that has a similar effect for those who pay and for those who do not pay. On the contrary, paying or not paying a bribe could pre-empt a rational choice, depending on the expected consequences shaped by firms’ characteristics and institutional and competitive environment.

The role of the institutional environment emerges as well as that of the competitive environment: a high level of competition is one of the main reasons firms turn to corruption. In this case, once corruption is chosen, it promotes innovative activities. On the contrary, in a weakly competitive environment, corruption seems useless because it does not generate benefits for the innovative activities.

The same holds true for the country effect, which is large and significant. In countries where corruption is a necessary condition to operate, firms practice bribery with positive effects on innovative activities. On the contrary, where corruption is not so important on a country-level, firms do not practice bribery as corruption would not increase (or may even decrease) innovative performance.

Building on these results, future research can now re-examine the well-established economic outcomes such as productivity as well as the economic performance impact of corruption, in the presence (or absence) of selection processes. More broadly, our findings suggest that corruption has highly complex consequences that transcend simple transactions between firms and officials. Bribery can act as an incentive as well as a tax for innovative activities, creating room for a rational adaptation of a firm’s strategies to a corrupt business environment.

Although this work is not without limitations, we believe it does provide a footpath for future research and related methods. Firstly, the cross-section nature of the BEEPS data prevents us from drawing any long-term implications of bribery strategies. Future research should focus on a more in-depth look at our conjecture by employing longitudinal datasets.

Secondly, although BEEPS stand out as one of the best sources of data for capturing firm-level corruption, it is vulnerable to measurement issues. Self-reported survey data on the incidence of corruption can substantially underestimate the actual prevalence of corruption (Kraay & Murrel, 2016). Biases pointing to the underestimates of corruption in transition and emerging markets are well recognized in the presence of informational asymmetries and reticence. This would suggest finding newer methods for reticence-adjusted estimates of corruption.

To go in depth into the multifaceted nature of corruption and its interactions on the micro, meso, and macrolevel, requires us to shift the microdata so as to overcome the shortcomings in cross-country macrodata and also to settle the correct way of measuring corruption in observational as well as in experimental data.

## Appendix

See Table A1.

**Table A1:** Number of firms per country

Country	Number of firms in the sample	Number of firms in sub-sample 1	Number of firms in sub-sample 2	Number of firms in sub-sample 3	Number of firms in sub-sample 4	Number of firms in sub-sample 5	Number of firms in sub-sample 6
Albania	360	360	280	232	17	17	230
Armenia	245	245	237	234	7	6	232
Azerbaijan	248	248	161	159	0	7	158
Belarus	285	285	258	257	20	20	250
Bosnia-Herzegovina	297	297	281	279	16	16	269
Bulgaria	273	273	214	211	17	16	200
Croatia	322	322	285	283	26	26	271
Czech Republic	217	217	196	193	14	12	170
Egypt	2457	2457	2149	2117	247	234	2050
Estonia	243	243	169	163	2	2	153
Macedonia	346	346	299	299	27	26	295
Georgia	289	289	275	274	3	3	270
Hungary	197	196	134	129	7	6	116
Israel	438	436	380	377	0	1	364
Jordan	548	540	466	445	13	13	422
Kazakhstan	430	430	312	306	44	43	296
Kosovo	179	179	154	151	29	29	145
Kyrgyzstan	215	214	174	171	78	75	165
Latvia	270	261	188	169	6	6	163
Lebanon	483	483	364	347	95	89	324
Lithuania	225	220	198	185	10	10	174
Moldova	312	311	260	250	9	9	239
Mongolia	324	324	256	250	50	49	243
Montenegro	102	102	85	85	9	8	82
Morocco	374	369	300	291	31	26	243
Poland	392	390	341	323	33	31	289
Romania	476	476	404	398	39	36	378
Russia	3030	3019	2386	2343	258	252	2262
Serbia	333	333	237	237	21	20	216
Slovak Republic	173	172	140	138	10	9	130
Slovenia	244	244	211	210	33	32	205
Tajikistan	253	253	177	173	32	29	167
Tunisia	580	580	405	405	5	4	376
Turkey	839	834	810	777	45	42	745
Ukraine	769	767	141	136	99	94	129
Uzbekistan	365	365	351	349	8	8	348
**TOTAL**	17,133	17,072	13,678	13,346	1360	130	12,769

- Subsample 1: Firms with non-missing data for *innoprod*.
- Subsample 2: Firms with non-missing data for *bribe* and *bribe_yes*.
- Subsample 3: Firms included in the estimations of columns 1 and 2 in Table 2.
- Subsample 4: Firms included in the estimation of column 3 in Table 2.
- Subsample 5: Firms included in the estimations of columns 6 and 9 in Table 2.
- Subsample 6: Firms included in the estimation of the endogenous switching model.
- The subsample of firms included in the estimations of column 4,5, 7 and 8 of Table 2 is almost identical to Subsample 6; the only difference is represented by 13 firms in Lebanon, which are included in the estimation of ESM and not included in these estimations, because sector is missing.

See Table A2.

**Table A2:** Firms by sector

Sector	Number of firms in the sample	Number of firms in sub-sample 3	Number of firms in sub-sample 4	Number of firms in sub-sample 5	Number of firms in sub-sample 6
Food	1567	1176	127	123	1103
Tabacco products	28	19	35	33	19
Textiles	473	389	66	60	371
Garments	869	629	17	14	593
Tanning and leather	172	154	22	22	143
Wood	363	292	22	22	281
Paper and paper products	56	42	6	6	39
Publishing, printing and recorded media	396	335	39	39	318
Coke and refined petroleum	6	5	0	0	4
Chemicals	445	387	26	24	376
Plastic and rubber	370	294	26	26	275
Non metallic mineral products	702	521	64	62	505
Basic metals	68	52	6	6	48
Fabricated metal products	685	552	63	59	526
Machinery and equipment	420	280	39	36	256
Office machinery	19	12	0	2	12
Electronics	187	154	18	18	147
Communication equipment	24	17	0	1	15
Precision instruments	145	116	11	11	112
Motor vehicles	76	52	5	4	49
Other transport equipment	31	25	0	3	23
Furniture	380	320	31	31	311
Recycling	28	17	4	4	16
Construction	1125	878	115	108	838
Services of motor vehicles	292	231	18	18	226
Wholesale	1878	1445	164	162	1402
Retail	3250	2539	207	201	2460
Hotel and restaurant	452	358	33	31	350
Transport	259	212	25	25	206
Supporting transport activities	203	157	23	22	151
Post and telecommunications	111	84	8	7	83
Information Technology	161	126	12	11	119
Other manufacturing	751	598	65	57	541
Retail/wholesale	249	196	20	17	187
Transport, supporting transports, post and telecommunications	114	103	11	11	102
Other services	558	424	29	29	398
Hotels	104	78	12	11	75
Restaurants	94	77	13	12	76
Others	2	0	0	0	0
**TOTAL**	17,113	13,346	1360	1306	12,756

- Sectors are identified according to the International Standard Industrial Classification code -ISIC Revision 3.1.
- Subsamples 3, 4, 5 and 6 are defined in the footnote of Table A1.
- For 13 firms in Lebanon belonging to Subsample 6 the sector is not defined (as the sector is not a variable included in the model, also firms with not defined sector may be included): therefore, the number of firms belonging to Subsample 6 is 12,769 but we could define the sector for 12,756 of them.
- Those 12,756 firms are the subsample for the estimations of column 4,5, 7 and 8 of Table 2.
- Note: the database presents some inconsistency and non-homogeneity in the classification of sectors, especially because of MENA-ES countries (Israel, Egypt, Jordan, Lebanon, Morocco, Tunisia). In these countries only a few sectors are identified: four in Israel (Food, Retail, Other manufacturing, Other services) and in Lebanon (Food, Other manufacturing, Retail/Wholesale, Other services), five in Tunisia, Morocco (Food, Garments, Retail, Other manufacturing, Other services) and in Jordan (Food, Garments, Other manufacturing, Wholesale/retail, Other services); in Egypt, more sectors are identified but many firms are classified in “Other manufacturing” and Transport, Supporting transports, Post and telecommunications are not distinguished; on the contrary, while for the other countries hotels and restaurants are included in the same category, in Egypt they are distinguished.

See Tables A3, A4 and A5.

**Table A3:** Correlations between innovation and corruption variables

	innoprod	innosales	corruption	bribes	bribes_yes
Innoprod	1.000				
Innosales	0.698**	1.000			
Corruption	0.091**	0.062**	1.000		
Bribes	0.051**	0.039**	0.542**	1.000	
bribes_yes	0.007	0.013**	–	1.000	1.000

**Table A4:** Innovation and corruption

	No informal payment (*bribe_yes* = 0)	Informal payment (*bribe_yes* = 1)	All firms
No product innovation	9304 (91.3%)	882 (8.7%)	10,186 (100%)
Product innovation	2936 (85%)	517 (15%)	3453 (100%)
Total	12,240 (89.7%)	1399 (10.3%)	13,639 (100%)

**Table A5:** Mean bribes between innovators and non innovators

	Mean of positive bribes	Mean bribes All firms
No product innovation	6.40	0.55
Product innovation	6.54	0.98
All firms	6.45	0.66

## Footnotes

1 Bardhan (1997); Rose-Ackerman Palifka, (2016). For alternative classification of corruption in the business perspective, see Cuervo-Cazurra (2016).

2 We are aware the two terms are often used for different purposes. For instance corruption is clearly distinguished from bribery by Chadee et al*.* (2021), as two distinct forms of informal institutions. Many types of payments or favors may be fairly or unfairly labeled as bribes, one of the best recognized type of corruption. One must be careful not to generalize social and cultural norms when examining bribery. Expectations of when a monetary transaction is appropriate can be very different from place to place.

3 The case of Siemens operating in countries characterized by different syndromes of corruption such as China; Russia, Italy and United States is examined by Kartner and Warner (2015). Some cases in the international management literature also suggest that the pervasiveness and arbitrariness of corruption can affect multinationals’ organizational legitimacy and strategic decision-making by substantially changing the modes of entry as shown by Rodriguez et al. (2005).

4 Endogenous-switching technique to explore micro-economic survey-based data from both innovating and non-innovating firms are applied to European firms by Crowley and McCann (2017). Interesting applications are found in different fields, for instance a selection model for climate change adaptation is applied by Di Falco et al. (2011).

5 Litvinova and Segnana (2018) show the local dimension of administrative corruption across Turkish regions.

6 BEEPS stands for Business Environment and Enterprise Performance Survey, V indicates the fifth survey. Countries are: Albania, Armenia, Azerbaijan, Belarus, Bosnia and Herzegovina, Bulgaria, Croatia, Czech Republic, Estonia, Georgia, Hungary, Kazakhstan, Kyrgyz Republic, Kosovo, Lithuania, Latvia, FYR Macedonia, Moldova, Mongolia, Montenegro, Poland, Russian Federation, Romania, Serbia, Slovak Republic, Slovenia, Tajikistan, Turkey, Ukraine, Uzbekistan. MENA-ES stands for Middle East and North Africa Enterprise Surveys. In this case countries considered here are: Egypt, Israel, Jordan, Lebanon, Morocco, Tunisia, a subsample of countries taken into consideration by MENA-ES.

7 For complete information about methodological aspects of the survey: https://www.beeps-ebrd.com/methodology/.

8 A survey of microeconometric estimation of treatment effects with special attention to heterogeneity is in Caliendo and Hujer (2005).

9 The split variable is the actual decision to corrupt or not: Eq. (2a) is estimated among the firms that actually paid a bribe, Eq. (2b) among the firms that actually did not pay a bribe.

10 The procedure runs as follows. Once estimated the coefficients (which are different in Regime 1 and Regime 2), applying the two outcome equations, for each firm two predicted values of the dependent variable are calculated, using respectively the parameters of Regime 1 and of Regime 2. The next step is to divide the whole sample in two groups: the firm that paid a bribe (the corrupting firms) and the firms that did not pay a bribe (the non-corrupting firms). Then, four averages are calculated (the four expected values illustrated in Table 1): (a) among the corrupting firms, the average of the predicted values obtained using the coefficients of Regime 1 (cell (a) of Table 1); (b) among the corrupting firms, the average of the predicted values obtained using the coefficients of Regime 2 (cell (c) of Table 1); (c) among the non-corrupting firms, the average of the predicted values obtained using the coefficients of Regime 1 (cell (d) of Table 1); (d) among the non-corrupting firms, the average of the predicted values obtained using the coefficients of Regime 2 (cell (b) of Table 1.

11 The unobserved factors affecting the corruption choice are correlated with the unobserved factors affecting innovation.

12 The independent variables affect the dependent variable in different ways in the two observed subsamples.

13 If the switching between the two different regimes is exogenous, σ_1η_ and σ_2η_ are equal to zero. In this case, the difference between TT and TU is given by (β1 – β2) (X_1i_–X_2i_), therefore it depends on the different amount of the observable determinants of the outcome in the two groups and on their different effect on the outcome in the two regimes. If the switch is endogenous, the difference is given by the previous effect plus the effect given by the correlation among the residuals of the outcome functions and the residuals of the selection equation.

14 Country and sector dummies have been excluded because the model is somewhat cumbersome. Thus, proxies for country effects have been utilized; checks for sector subsamples show that results are not driven by sector specificities. Results are available upon request.
